# Evaluation of Bioactive Restorative Materials’ Color Stability: Effect of Immersion Media and Thermocycling

**DOI:** 10.7759/cureus.43038

**Published:** 2023-08-06

**Authors:** Roaa Abuljadayel, Ali Mushayt, Talal Al Mutairi, Shara Sajini

**Affiliations:** 1 Restorative and Aesthetic Dentistry, Faculty of Dentistry, King Abdulaziz University, Jeddah, SAU; 2 General Dentistry, Faculty of Dentistry, King Abdulaziz University, Jeddah, SAU

**Keywords:** spectrophotometer, thermocycling, staining, glass ionomers, resin composite, giomers, bioactive materials

## Abstract

Objectives: One of the most important aspects that determines the clinical lifetime of aesthetic restorations, is the color stability (CS) over the long term. This study aims to assess the effect of artificial aging and thermocycling in different staining solutions on the CS of bioactive restorative materials compared to conventional ones.

Methods: The following four material groups were investigated: ACTIVA™ Bioactive (AB) (Pulpdent Corporation, Massachusetts, United States), Beautifil II (BF) (SHOFU Inc., Kyoto, Japan), Fuji II (FJ) (GC Corporation, Tokyo Japan), and Filtek™ Z350 XT (FT) (3M, Minnesota, United States). A total of 100 specimens were fabricated using an acrylic mold and then immersed in five staining solutions groups: coffee, black tea, Cola, mixed berry juice, and saline. Baseline shade (T0) was recorded using two spectro-photometer devices, VITA Easyshade® Advance (VITA Zahnfabrik, Baden-Wurttemberg, Germany) and Color-Eye® 7000A (X-Rite, Inc., Grand Rapids, Michigan, United States). Following this, the shade was recorded at 30 days (T1), three months (T2), and after 5000 thermocycles (5C-55C) (T3). CIE L*a*b* (International Commission on Illumination color space) data was used to calculate ΔE for each group.

Results: All samples showed a significant color change (P<0.001) following one and three months of storage and after thermocycling. AB and BF showed the highest CS over time, whereas FT and FJ showed the least CS.

Conclusion: The CS of restorative materials is mostly determined by their type, followed by staining solution type and thermocycling. The most potent solutions were coffee, tea, and berry juice.

## Introduction

Tooth-colored restorative materials have been designed to restore aesthetics and function as well as easy bonding to both enamel and dentin. They were formulated to have a passive effect on the tooth. In recent years, dental restorative materials are tending to be more bioactive, which means that they can interact with the body in different ways. Bioactive materials have had a great impact on restorative dentistry, improved the longevity of restoration, and helped in the prevention of recurrent caries. These materials can remineralize the tooth structure by fluoride exchange such as glass ionomer cements and giomers. Another form of activity can be shown as an apatite-like formation on the material surface when it interacts with body fluids or simulated body fluids (SBF) over time such as calcium phosphate and calcium silicate-based materials. Also, they can biologically interact with tooth structure to promote tissue regeneration [1].

The minimal invasive dentistry approach advocates the use of biocompatible and bio-interactive materials to preserve the tooth structure and to be able to remineralize and regain the mechanical properties of the tooth. In 1960, Wilson and Kent introduced glass ionomer cement (GIC) material, a water-based restorative material with anti-cariogenic properties that release fluoride, chemically bond to the tooth structure by forming polyalkenoate salts and favoring dentinal repair. Several modifications were followed to improve their mechanical properties and resin-modified glass ionomers (RMGICs) were introduced. RMGICs possess improved clinical properties. Moreover, they are self-adhesive materials that contain methacrylate-based monomers. They can be used in deeper cavities to minimize the stress concentration at the dentin-bonded interface [2]. Composite resins are a mixture of resin, fillers, initiators of the chemical reaction, and the coupling agent. The ratio of these combinations determines the properties of the produced material [3]. By combining the best properties of composite resins and glass-ionomers, giomers have been produced, by incorporating particles of pre-reacted glass filler in the matrix of the composite material, which can protect against caries and restore the tooth function and esthetics. It has been recommended by manufacturers that giomers can restore all cavities including high caries risk patients because of fluoride release, high aesthetic properties, and chameleon effect [4].

More recently, a bioactive resin-based restorative GIC has been formulated, which is composed of a combination of bioactive resin matrix with polyacid along with fluoro-alumino silicate particles; called ACTIVA™ Bioactive (AB) (Pulpdent Corporation, Massachusetts, United States). AB materials are made from a unique ionic resin matrix that releases calcium, phosphate, and fluoride ions when they come into contact with saliva [5]. These ions help to stimulate the natural remineralization process of the teeth, which can help to strengthen and protect them from decay. One of the key benefits of AB materials is that they are highly versatile and can be used for a wide range of dental applications.

The most crucial aesthetic components of a restoration are surface irregularity, surface shine, and color [6]. The ability of a material to retain its original color is one of the crucial factors that indicate the durability of tooth-colored restorations. Mimicking the shade of tooth-colored restoration with the adjacent teeth is essential for esthetic results and for lifetime durability. The dynamic oral environment may compromise the color stability of these materials. Tooth-colored restorations can be discolored by intrinsic or extrinsic factors. Intrinsic staining may generate due to physiochemical discoloration reactions within the material matrix [7]. In composite restoration, chemical discolorations may be attributed to oxidation of unreacted methacrylate group or polymer matrix. An unconverted camphorquinone residue causes a yellowish shift in light-activated resin composites when curing is insufficient. Moreover, tertiary amines, another component of the light-activated system, have a propensity to produce a yellowish or brownish stain in response to stimuli of "light" or "heat."

On the contrary, extrinsic staining is usually related to external surface changes such as the adsorption of plaque and stains, and exposure to environmental factors such as heat, water, and food colorants [8]. Besides this, it has been found that beverages (such as coffee, tea, and wine), smoking, physiochemical stress, and some foods can affect the surface of aesthetic restorative materials. These effects are primarily brought on by the absorption or adsorption of coloring substances found in food and beverages. On the other hand, diets and drinks cause the composite surface to superficially deteriorate, which lowers its hardness and roughness and causes external coloring. Various types of food colorants and other coloring agents have been studied over the years, and it was concluded that tea and coffee, beverages, grape juice, cherry juice, soya sauce, nicotine, and disinfecting agents used in mouth rinses have the potential to cause color changes of dental materials [9]. Other dietary factors that can cause composite discoloration include the consumption of highly acidic foods such as citrus fruits, as the acid can weaken the bond between the composite and the tooth surface, making it easier for stains to penetrate [10].

Since color perception differs from one person to another and might be expressed differently. Hence, color assessment tools were activated. The CIE L*a*b* (International Commission on Illumination color space) system measures color based on three parameters: lightness or brightness (L*), redness or greenness (a*), and yellowness or blueness (b*). A spectrophotometer is used to measure the reflectance or transmittance of light at different wavelengths, which helps calculate the values of L*, a*, and b* for a sample. These values can then be plotted on a three-dimensional graph to create a color space, with the CIE L*a*b* color space being the most commonly used [11-13]. 

The color stability of dental materials is an essential parameter for the aesthetic success of dental restorations, especially anterior teeth. Discoloration may be caused by several factors, such as food and drink consumption, oral hygiene products, and aging. Therefore, it is crucial to investigate the effect of different staining solutions, storage time, and materials on the color alteration of dental restorative materials. Numerous researches have evaluated the color stability of conventional dental restorations submerged in various solutions, but little is known about how this immersion combined with thermocycling affects the color stability of bioactive dental restorations.

This study aimed to evaluate the effect of thermocycling on color stability (∆E) of four different dental materials, Filtek™ Z350 XT (FT) (3M, Minnesota, United States), Fuji II (FJ) (GC Corporation, Tokyo Japan), Beautifil II (BF) (SHOFU Inc., Kyoto, Japan), and AB (Pulpdent Corporation), after immersion in five different staining solutions (coffee, tea, cola, berry, and saline) using two color measuring devices. The null hypotheses tested were that: (i) there was no difference between the two-color measuring devices; (ii) there was no difference in color change between materials groups; (iii) there was no difference in color change among different staining solutions; (iv) thermocycling didn’t affect the color stability of all groups.

## Materials and methods

This study was carried out in the Faculty of Dentistry, King Abdulaziz University, Jeddah, Saudi Arabia, after being reviewed and approved by the Research Ethics Committee at King Abdulaziz University (approval number: 160-12-20). It was conducted according to the guiding principles for investigational methods found in the Declaration of Helsinki of the World Medical Association.

Specimen preparation

Four types of restorative materials: AB (Pulpdent Corporation), BF (Shofu Inc.), FJ (GC Corporation), and FT (3M) were used in this study. The details of each material as given by their respective manufacturers are shown in Table 1. 

**Table 1:** Summary of the materials used in the study TEGDMA: triethylene glycol dimethacrylate; Bis-GMA: bisphenol A-glycidyl methacrylate; UDMA: urethane dimethacrylate; Bis-EMA: bisphenol A diglycidyl methacrylate ethoxylated; PEGDMA: poly(ethylene glycol) dimethacrylate; RMGIC: resin-modified glass ionomer

Materials	Types	Shade	Composition	Manufacturer
ACTIVA™ Bioactive	Reinforced Compomer	A1	Mixture of methacrylates and diurethane with amorphous silica (6.7%), modified polyacrylic acid (44.6%), and sodium fluoride (0.75%).	Pulpdent Corporation, Massachusetts, United States
Beautifil II	Giomer	A1	Bis-GMA, TEGDMA, aluminum oxide, silica, Aluminofluoro- borosilicate glass filler, pre-reacted glass-ionomer filler, camphoroquinone	SHOFU Inc., Kyoto, Japan
Fuji II	RMGIC	A1	58 wt% fluoro-aluminumsilicate, methacrylate, hydroxyethyl, polyacrylic acid, and water.	GC Corporation, Tokyo, Japan
Filtek™ Z350 XT	Nano-filled composite	A1	Fillers: silica cluster and Zirconia (20 nm). Matrix: Bis-GMA, UDMA, Bis-EMA, PEGDMA, and TEGDMA	3M, Minnesota, United States

The sample size was calculated following a pilot study, using a 0.05 alpha value and 80% power to detect a difference of 25%. A total of 100 disc-shaped samples were prepared, which were assigned into four groups according to materials (n=25). Each group was subdivided into five groups (n=5) according to the staining solution. Twenty-five discs (10 mm in diameter and 1 mm in thickness) from shade A1 of each resin material were fabricated by placing the material in a custom acrylic mold that was placed on a glass microscopic slide that was rested on a glass slab. The material was then adapted to the mold and covered with mylar strip and gently pressed by another glass slide to remove excess. Then, a light-emitting diode (LED) curing unit (Elipar™ FreeLight 2 LED Curing Light; 3M) was utilized to light cure each material's top surface at 1200 mW/cm^2^ power density, following the manufacturer's directions for each material. After polymerization, samples were polished with 1200-grit carborundum papers using a MetaServ™ 250 grinder-polisher (Bühler Holding AG, Uzwil, Switzerland) with Vector™ Power Head (Bühler Holding AG) under running water. [14-17].

Staining protocol

Five specimens from each group were placed in each of the following solutions: 5 mL coffee (Nescafe; Nestlé S.A., Vaud, Switzerland) (pH 5.1), 5 mL black tea (Lipton Black Tea; Lipton Teas and Infusions, Rotterdam, Netherlands) (pH 5.5), 5 mL cola (Coca-Cola; The Coca-Cola Company, Atlanta, Georgia, United States) (pH 2.7), 5 mL mixed berry juice (Almarai Mixed Berry Juice; Almarai Company, Riyadh, Saudi Arabia) (pH 4.5), and 5 mL saline (Pharmaceutical Solutions Industry Ltd, Jeddah, Saudi Arabia) (pH 7). All samples were stored in an incubator (Memmert GmbH + Co.KG, Schwabach, Germany) at 37°C for one and three months. During the staining storage period, staining solutions were replaced weekly with standardized concentrations and temperatures [18]. After that, all samples underwent thermocycling.

Thermocycling

The samples were divided into five subgroups according to the staining solution (coffee, tea, cola, berry, and saline group) within separate glass vials. All samples were aged artificially by the thermocycler (SD Mechatronik GmbH, Germany) in separate water chambers of 5°C and 55°C with a dwell time of 30 seconds and a transfer time of 15 seconds for 5000 cycles. Following the aging process, all samples were removed from beverages and rinsed with distilled water then dried with an absorbent paper. After that, the final color remeasurements were taken for each group as described above [19-21].

Color measurement

The spectrophotometer was calibrated prior to use using the calibration plate provided. All specimens’ shades were recorded at the baseline initially before immersion in staining solutions using two spectrophotometer devices: VITA Easyshade® Advance (VITA Zahnfabrik, Baden-Wurttemberg, Germany) against white calibration plate and Color-Eye® 7000A (X-Rite, Inc., Grand Rapids, Michigan, United States) against black calibration plate. In the VITA Easyshade digital device, the measurement tip was positioned perpendicular to the sample surface in direct contact to measure the characteristics of a specimen's color. The measuring point was consistently placed on each specimen using a positioning jig. The mean values of each sample's three measurements were used for analysis. The X-Rite spectrophotometer device was also calibrated for reflectance mode with a 6 mm aperture plate according to the manufacturer’s instructions prior to measurements.

Color measurement was conducted at the baseline (T0), following storage in staining solutions for one month (T1), three months (T2), and after thermocycling (T3). After removal from all solutions, the specimens were rinsed with distilled water and transferred to separate vials filled with saline and were placed in an ultrasonic cleaner (Powersonic 405, Hwashin Technology Co. Ltd, Gyeongsangbuk-do, South Korea) for five minutes then dried for 10 seconds before color measurement. The color changes were determined using the CIE L*a*b* system [22,23], and then they were used for ΔE calculation for each time point based on the equation below [24]:

∆E=√((L_post-L_baseline )^2+(a_post-a_baseline )^2+(b_post-b_baseline )^2 )

Statistical analysis

The mean and standard deviation values were calculated for each group in each test. Data were explored for normality using Kolmogorov-Smirnov and Shapiro-Wilk tests and showed parametric (normal) distribution. The paired sample t-test was used to compare between two groups in related samples. One-way ANOVA followed by the Tukey post hoc test was used to compare more than two groups in non-related samples. Independent sample t-test was used to compare two groups in non-related samples. The significance level was set at P ≤ 0.05. Statistical analysis was performed with IBM SPSS Statistics for Windows, Version 20.0 (Released 2011; IBM Corp., Armonk, New York, United States).

## Results

Color stability (∆E)

**Figure 1:**
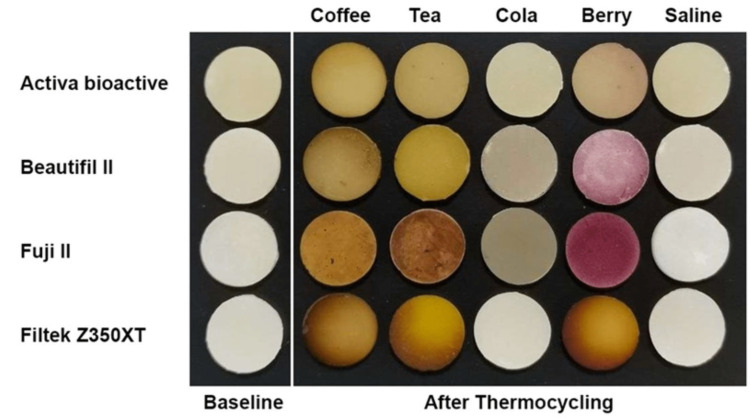
Visual changes of different materials groups between baseline and after thermocycling in different staining solutions Material Groups: ACTIVA™ Bioactive (Pulpdent Corporation, Massachusetts, United States); Beautifil II (SHOFU Inc., Kyoto, Japan); Fuji II (GC Corporation, Tokyo Japan); and Filtek™ Z350 XT (3M, Minnesota, United States) Staining Solutions: coffee (Nescafe; Nestlé S.A., Vaud, Switzerland); black tea (Lipton Black Tea; Lipton Teas and Infusions, Rotterdam, Netherlands); cola (Coca-Cola; The Coca-Cola Company, Atlanta, Georgia, United States); mixed berry juice (Almarai Mixed Berry Juice; Almarai Company, Riyadh, Saudi Arabia); saline (Pharmaceutical Solutions Industry Ltd, Jeddah, Saudi Arabia)

**Figure 2:**
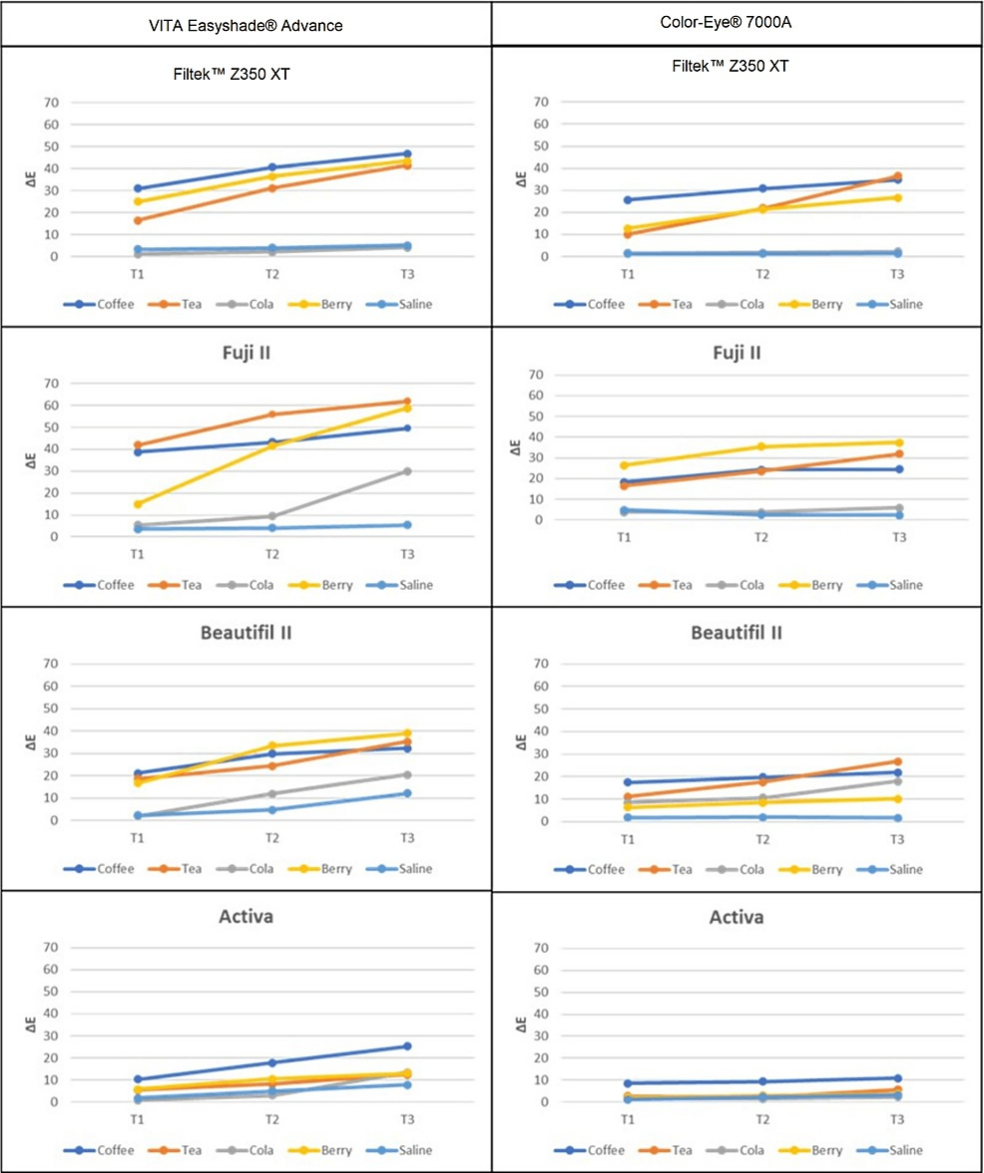
Line graphs showing ΔE values from the two spectrophotometer devices for the four tested materials after storing in different solutions for one month (T1), three months (T2), and after thermocycling (T3). Spectrophotometer Devices: VITA Easyshade® Advance (VITA Zahnfabrik, Baden-Wurttemberg, Germany); Color-Eye® 7000A (X-Rite, Inc., Grand Rapids, Michigan, United States) Tested Materials: ACTIVA™ Bioactive (Pulpdent Corporation, Massachusetts, United States); Beautifil II (SHOFU Inc., Kyoto, Japan); Fuji II (GC Corporation, Tokyo Japan); and Filtek™ Z350 XT (3M, Minnesota, United States) Staining Solutions: coffee (Nescafe; Nestlé S.A., Vaud, Switzerland); black tea (Lipton Black Tea; Lipton Teas and Infusions, Rotterdam, Netherlands); cola (Coca-Cola; The Coca-Cola Company, Atlanta, Georgia, United States); mixed berry juice (Almarai Mixed Berry Juice; Almarai Company, Riyadh, Saudi Arabia); saline (Pharmaceutical Solutions Industry Ltd, Jeddah, Saudi Arabia)

All samples showed visual changes in color after one month (T1) (Figure 1). Repeated measure ANOVA was used to compare more than two groups in related samples. The results showed that storage time, materials, and staining solutions have a statistically significant (p < 0.001) effect on ∆E. The strongest effect of staining was found to be from coffee followed by mixed berry juice and tea across all groups, except the FJ group, which was the most significantly affected group by mixed berry juice (p<0.001) (Figures 1, 2). All ∆E values of all staining solutions were significantly different from each other (p<0.001). Nevertheless, the effect was mainly dependent on the ‘type of material’ followed by ‘staining solution type’ and ‘storage time’. Multiple comparisons of the material effect for both devices showed that the FJ group recorded significantly higher ∆E values (∆E value after thermocycling = 61.83; p < 0.001) compared to other groups. The least affected material was the AB group followed by the BF group. The ‘storage time’ had a significant effect starting from T1, and then significantly increased at T3 (p<0.001) (Figure 1, Table 2). Mean values for color changes at T2 and T3 were reported (Table 2). From both color-measuring devices, there were comparable results where p-values were compared between the two devices within each group. However, Vita Easyshade resulted in a higher ΔE value than the X-Rite spectrophotometer in all groups (p<0.001) (Figure 1, Table 2). Significant changes were found when comparing the devices in the same storage media.

**Table 2:** Means and standard deviations of the ∆E values from two spectrophotometer devices in different material groups after thermocycling in different staining solutions Spectrophotometer Devices: VITA Easyshade® Advance (VITA Zahnfabrik, Baden-Wurttemberg, Germany); Color-Eye® 7000A (X-Rite, Inc., Grand Rapids, Michigan, United States) Material Groups: ACTIVA™ Bioactive (Pulpdent Corporation, Massachusetts, United States); Beautifil II (SHOFU Inc., Kyoto, Japan); Fuji II (GC Corporation, Tokyo Japan); and Filtek™ Z350 XT (3M, Minnesota, United States) Staining Solutions: coffee (Nescafe; Nestlé S.A., Vaud, Switzerland); black tea (Lipton Black Tea; Lipton Teas and Infusions, Rotterdam, Netherlands); cola (Coca-Cola; The Coca-Cola Company, Atlanta, Georgia, United States); mixed berry juice (Almarai Mixed Berry Juice; Almarai Company, Riyadh, Saudi Arabia); saline (Pharmaceutical Solutions Industry Ltd, Jeddah, Saudi Arabia)

Materials		Solutions		VITA Easyshade® Advance						Color-Eye® 7000A				
				Mean		SD		P-Value		Mean		SD		P-Value
ACTIVA™ Bioactive	Coffee		25.24		± 3.61		0.002*		10.83		± 1.83		0.358ns	
	Tea		12.48		± 1.66		0.011*		5.6		± 0.94		0.003*	
	Berry		12.99		± 2.69		0.008*		3.62		± 0.39		0.063ns	
	Cola		13.47		± 2.65		0.001*		2.33		± 0.2		0.013*	
	Saline		7.9		± 2.07		0.013*		3.09		± 0.56		0.002*	
Beautifil II	Coffee		32.34		± 2.22		0.054ns		21.92		± 1.29		0.028*	
	Tea		35.27		± 3.61		0.005*		26.65		± 3.24		0.026*	
	Berry		38.99		± 6.84		0.186ns		10.08		± 2.7		0.324ns	
	Cola		20.47		± 3.75		0.003*		17.95		± 3.75		0.011*	
	Saline		12.22		± 1.71		<0.001*		1.68		± 0.36		0.415ns	
Fuji II	Coffee		49.66		± 4.9		0.004*		24.46		± 5.13		0.001*	
	Tea		61.83		± 7.14		0.052ns		31.81		± 4.01		<0.001*	
	Berry		58.77		± 1.26		<0.001*		37.32		± 4.06		0.009*	
	Cola		29.98		± 3.26		<0.001*		5.84		± 1.62		0.080ns	
	Saline		5.46		± 0.82		0.030*		2.37		± 0.55		0.897ns	
Filtek™ Z350 XT	Coffee		46.82		± 7.55		0.027*		34.87		± 3.45		0.013*	
	Tea		41.54		± 6.59		0.029*		36.53		± 2.41		<0.001*	
	Berry		43.54		± 4.22		0.034*		26.59		± 4.05		0.035*	
	Cola		4.11		± 0.74		0.003*		2.25		± 0.68		0.138ns	
	Saline		5.15		± 0.78		0.102ns		1.46		± 0.25		0.357ns	

## Discussion

Bioactive materials are designed to release bioactive ions that can stimulate the formation of hydroxyapatite, the mineral that makes up tooth enamel and dentin. By promoting the formation of hydroxyapatite, bioactive restorative materials can help to repair and strengthen damaged or decayed teeth. One of the key benefits of bioactive restorative materials is their ability to promote the natural healing and remineralization of teeth, which can help to prevent the need for more invasive treatments. In addition, bioactive restorative materials can provide excellent bonding and sealing properties, which can help to protect teeth from further damage [25,26], However, their color stability can be affected by several factors including their composition, fabrication, and the environment in which they are placed. The oral environment's dynamic character, with frequent variations in pH, stress, and temperature, may have a major impact on the color stability of aesthetic and bioactive restorative materials. Every day, the oral environment is exposed to a wide range of media, many of which have the potential to stain or otherwise change the surfaces of dental restorations, potentially degrading their aesthetic quality. It is well known that dietary factors have an impact on the staining of oral tissues and restorations. The current investigation revealed considerable changes in the color characteristics of some tested materials after thermocycling.

VITA Easyshade and Color-Eye 7000A are both commonly used tools for measuring and matching the color of dental restorations. However, there are some differences between the two tools. While both spectrophotometers are effective tools for color measurement of dental restorations, X-Rite spectrophotometer (Color-Eye 7000A) are generally considered to be more accurate and precise, while VITA Easyshade is designed for ease of use in clinical settings [13]. The result of the current study demonstrated that there was a significant difference between the two-color measuring devices which reject the first hypothesis. However, these differences didn’t affect the results. These variations were discovered when both devices were compared within each group. However, these differences didn’t impact the fact that AB and BF have superior color stability than FJ (GIC) and composite materials after thermocycling. Furthermore, both devices supported that storage time and change in temperature had an influence on the color stability of the materials regardless of the staining solution used. As a result, both devices produced different ∆E values, although this had no effect on the comparison of the color stability performance of the tested materials in this study. Similar results were noted in a previous study reporting the differences between the devices [18].

Several studies have investigated the color stability of bioactive materials, both in vitro and in vivo. Previous investigations concluded that the type of restorative material has a significant impact on its color stability. Xu et al. evaluated the color stability of four different bioactive materials after exposure to artificial saliva and various staining solutions. All materials experienced some degree of color change after storage, but the degree of color change varied between the materials and the type of staining solution used [27]. The findings of this study also indicate that the type of material had the greatest impact on color stability, followed by staining solution type and storage time. Among the materials tested, FJ recorded significantly higher ∆E values compared to other groups, indicating that it was more susceptible to color changes over time and after thermocycling. This is consistent with a previous study that has shown that GIC materials are more susceptible to discoloration than composite materials [6]. Another study evaluated the color stability of two different bioactive materials following coffee exposure and thermocycling. Both materials changed in color, but the bioactive composite resin showed greater color stability than the RMGIC. These results agree well with the results of the current study, which rejects the second null hypothesis as there was significant difference in color between materials groups.

The resin matrix, size of the filler particles, degree of polymerization, and staining agents are all factors that affect color stability. The relatively new bioactive materials in this study were AB and BF, both materials showed resistance to color changes after thermocycling in most of the staining groups (except berry and tea in the BF group) s compared to FJ and composite groups. The chemical composition and filler particles, as well as the smooth surface finish of AB and giomer (BF) materials may contribute to their high resistance to color change. AB is a bioactive composite material that contains an ionic resin matrix, increasing filler loading, which in turn reduces the water sorption and solubility of the material, which helps to prevent staining and discoloration [28].

Giomer materials are also designed to be highly resistant to staining and discoloration due to their unique chemical composition, which includes a combination of glass fillers and a special resin matrix [11]. In addition, the smaller particle size of 0.8 m and higher filler loading of 83.3 wt% of giomer which leads to a smoother surface has led to fewer surface stains retained by giomer with a smaller particle size than rough surfaces. Additionally, the considerably increased triethylene glycol dimethacrylate (TEGDMA) concentration may contribute to the reduced staining susceptibility. [29]. On the other hand, GIC (FJ) recorded the highest color change, which may be attributed to the high porosity of RMGIC materials which may allow greater penetration of staining agents. In agreement with the results of this study, Alrahlah et al. evaluated the color stability of several restorative materials, including Fuji II LC, after immersion in black tea and blueberry juice with and without thermocycling. The results showed that Fuji II LC exhibited the highest color change after immersion in both solutions [30].

Common beverages were used in the current study such as coffee, tea, cola, and berry juice which are commonly used throughout the world. The results of this study also showed that color stability, as measured by ∆E values, was significantly affected by staining solution. The staining solutions that had a significant effect on color stability were coffee, tea, and berry, which reject the third hypothesis. However, there were no significant differences between these solutions in terms of their effect on ∆E values. This is consistent with previous studies that have shown that coffee, tea, and berry are common dietary sources of staining agents that can cause discoloration of dental materials. Of all the tests, coffee and berry juice then tea had the strongest impact on color stability. Coffee produced darker samples due to significant variations on all axes (L*, a*, and b*) (Figure 2). Increased a* had the greatest positive impact on the color of mixed berry juice, resulting in reddish samples. Contrarily, tea changes mostly on b*, giving the samples a more yellowish color. The results of this study are in alignment with previous studies in which coffee caused the greatest color alteration, whereas cola had the lowest color alteration effect [15,17]. Temperature and acidity are two parameters belonging to staining solutions that may contribute to the observed color change in the examined material. Both elements can have a major impact on the color stability of restorative materials, resulting in an unappealing esthetic appearance and necessitating restoration replacement. Therefore, these factors were standardized during the experiment to exclude their effect. Furthermore, the solution was changed every week with a new beverage to simulate the ongoing intake of these beverages.

Thermocycling is a process that involves subjecting dental materials to alternating cycles of high and low temperatures to mimic the conditions of the oral environment. This process is commonly used to evaluate the performance of dental materials, in terms of their resistance to thermal stress and their ability to maintain their properties over time. According to the results of this study, all examined materials had color changes after storage for one month and three months, and after thermocycling. The staining medium and the type of material both had an impact on the color stability of all examined materials. All samples also exhibited visual changes starting from week 4, and these changes continued to increase up to three months, and after thermocycling, which rejects the fourth hypothesis. This is consistent with previous studies that have shown color alterations in dental materials due to exposure to staining solutions over time [6].

Poggio et al. evaluated the color stability and surface roughness of three different bioactive materials (two GIC and one RMGIC) after exposure to coffee and red wine staining solutions and thermocycling. They concluded that the degree of color change varied between the different materials with the most change in RMGIC [6]. It is worth noting that dental materials exposed to staining solutions over a prolonged period are at a greater risk of color changes. Additionally, manufacturers should continue to develop dental materials with improved color stability to minimize the risk of discoloration. However, it is important to note that many factors can influence the color stability of dental materials, including the specific type of solution used, the duration and frequency of exposure, and the method of evaluation. Therefore, these results should be interpreted with caution, and additional studies may be needed to fully understand the color stability of restorative materials in clinical settings.

## Conclusions

According to the results of the current study, the color stability of restorative materials is mostly determined by their type, followed by staining solution type and thermocycling. Coffee was the most effective staining solution, followed by tea and mixed berry juice. Furthermore, when compared to other traditional restorative materials, the AB and BF materials demonstrated promising color stability findings. Despite discrepancies between the values of the two devices, both spectrophotometers (VITA Easyshade and X-Rite Color-Eye) produced comparable results for the color stability of the materials. The findings of this study have important implications for dental practitioners and manufacturers. The results suggest that the type of material used for restorations can significantly impact color stability, and practitioners should consider this when selecting materials for their patients.
